# Parental stress when caring for a child with cancer in Jordan: a cross-sectional survey

**DOI:** 10.1186/1477-7525-10-88

**Published:** 2012-07-28

**Authors:** Rami Masa’Deh, Jacqueline Collier, Carol Hall

**Affiliations:** 1 School of Nursing, Midwifery & Physiotherapy, University of Nottingham, Queen's Medical Centre, Nottingham, NG7 2HA, UK; 2 School of Allied Health Professions, University of East Anglia, Queen's Building, Norwich Research Park, Norwich, NR4 7TJ, UK; 3 University of Nottingham, School of Nursing, Midwifery & Physiotherapy, Queen's Medical Centre, Nottingham, NG7 2HA, UK

**Keywords:** Parents, Children, Cancer, Stress, Caregivers, Mothers, Fathers

## Abstract

**Background:**

Most studies report that being parents of a child with cancer is a stressful experience, but these have tended to focus on mothers and few have included both parents. Moreover, studies have focussed on families in Western countries and none have been published examining the psychological outcomes for parents living in an Arabic country.

This research explores the stress levels of Jordanian parents caring for a child with cancer in order to identify the psychological needs of parents in this environment and to explore how mothers and fathers stress levels might differ.

**Methods:**

The study was carried out in Jordan using the Perceived Stress Scale 10-items (PSS10). The questionnaire was completed by 300 couples with a child who has cancer and a comparison group of 528 couples where the children do not have any serious illness. Multivariate backward regression analysis was carried out.

**Results:**

Analysis adjusting for spousal stress and sociodemographic predictors revealed that stress levels of mothers with a child who had cancer remained significantly higher than mothers whose children did not have any serious illness (p < 0.001). However, having a child with cancer did not show a significant association with the fathers’ reported stress scores (p = 0.476) when spousal stress was in the model, but was highly significant once that was removed (p < 0.001).

Parental stress was analysed for those with a child who has cancer and in models which included spouse’s stress scores, sociodemographic and cancer-related predictors 64 % of the variance was explained for mothers (adjusted R^2^ = 0.64, p < 0.001) and fathers (adjusted R^2^ = 0.64, p < 0.001). Models excluding spousal stress scores explained just 26 % of the variance for fathers and 22 % for mothers.

**Conclusions:**

This is the first study into the psychological outcomes for parents living in an Arabic country who care for a child with cancer. Both mothers and fathers with a child diagnosed with cancer reported higher stress levels than those from the normal Jordanian parent population. Mothers and fathers of children with cancer reported significantly different levels of stress to each other but models reveal significant contributions of the stress score of fathers upon mothers, and vice versa. The findings provide evidence of the need for psychological support to be developed for families caring for a child with cancer in Jordan.

## Background

The number of children with cancer is rising, caused by increases not only in incidence but also in survival rates. The incidence rate of childhood cancer in the United States has increased from about 11.5 cases per 100,000 children in 1975 to 14.9 per 100,000 children in 2004 [1]. The incidence rate of childhood cancer in Jordan fluctuated between 9 and 10 cases per 100,000 children between the period of 2000 and 2007 [2]. Different methods are used to treat cancer. Chemotherapy; radiotherapy; surgery; gene therapy; bone marrow transplant; and a combination of any or all of these are ways to treat childhood cancers [3]. Side effects of these treatment options are varied and depend on the patient’s age, type of cancer, type of treatment, etc. [4]. The aggressive nature of cancer treatment methods and their side effects e.g. nausea, vomiting, hair loss, weight change, mood change, general weakness, low immunity and adverse effect on reproductive system and possibility of infertility have been highlighted [4]. Moreover, there is a possibility of developing a secondary cancer as a result of radiotherapy treatment e.g. secondary bone cancer seen in childhood cases treated with radiotherapy [5].

Although adverse effects for childhood cancer treatment are widely acknowledged, the significant progress in the treatment options of childhood cancers for the last few decades is seen in the increase in survival rate. The overall survival rate for paediatric cancer for the 5 years after diagnosis in the United Kingdom, for the year 2000, is about 70 % for all paediatric malignancies [6] compared with a 25 % survival rate for all paediatric cancer in 1970 [7]. Survival rate improvements in childhood cancer patients have changed the nature of the disease from being a fatal disease to frequently becoming a chronic one. This increase in the number of children with cancer gives rise to increasing numbers of parents who are caring for such children.

Most paediatric oncology literature agrees that being a parent of a child with cancer is an emotionally stressful event [8-16]. Previous studies have showed that parents of a child with cancer are involved in a long term therapy with their child [17]. They may live with continuous uncertainty about the outcomes [18], and they may have to live with the threat of relapse or death for years [8]. They often have to change their family daily life and some of their roles and responsibilities [18-20]. Parents have been found to be psychologically affected by their child’s diagnosis, time since diagnosis, side effects of the treatment and child’s health status [10]. Additionally, parents have reported burden in their employment and financial status [21-24], their family relationships [8], caring for the other children [19] and they feel guilty sometimes [18,25]. Although these stressors were found to affect mothers and fathers parenting children with cancer, mother and fathers were found to have different levels of perceived stress [26,27]. Klassen et al. [28] carried out a systematic review into the factors related to parental health and wellbeing when caring for a child with cancer and identified that five main areas were found to contribute to psychological health of the parents: family social and economic characteristics, child characteristics, caregiver demands, self perception, and coping.

Most of the previous studies investigating stressors in parents of a child with cancer were conducted in Western communities (e.g. United Kingdom, United States, Greece, Netherlands, Switzerland, etc.) with none in the Arab world and no studies were found in Arabic language. The results of these Western studies cannot necessarily be generalised to parents of children with cancer across different ethnicities or cultural backgrounds and there is a need for research in further settings. This research aimed to examine the topic within the Jordanian population. Jordan is a relatively small country situated in the Middle East, with Amman as the capital city. According to the Jordanian Department of Statistics [29], the total population of Jordan is about 5.85 million people and approximately 75 % live in the three main cities (i.e. Amman, Zarqa and Irbid). Arab people are the largest ethnic group in Jordan (98 % of total population) and 92 % are Muslim [30]. Jordanian families experience stable home environments with most children living with both parents. Less than 0.5 % of the child population are not living with their biological parents [31].

Health care services in Jordan are highly developed and are viewed as among the most modern in the Middle East, comparing favourably to other developed countries. Many people from different countries around Jordan depend on Jordan for medication and treatment [32]. In 1994, Jordan’s health care expenditure amounted to approximately 7.9 % of its gross domestic product [33]; increasing to 9.1 % by 2007 [34]. In Jordan, there are three major hospitals that provide cancer treatment to Jordanian patients. These hospitals reflect the three healthcare sectors found in Jordan; public healthcare, private healthcare and military healthcare. However, there is little or no formal psychological support for parents within the healthcare setting. Understanding and knowledge of the parental experiences of having a child with cancer in an Arab Muslim community is not clear and has not been a focus of research.

This research will be the first to report on the stress within couples caring for a child with cancer in an Arabic setting and the aim of the proposed research is to explore mothers’ and fathers’ stress levels when having a child with cancer in Jordan and to compare their stress levels with Jordanian parents who do not have children with any serious illness.

## Methods

The cross-sectional survey was carried out in Jordan using an Arabic version of the Perceived Stress Scale 10-items (PSS10). The questionnaire was completed by 300 couples with a child who has cancer and a comparison group of 528 couples where the children do not have any serious illness. Multivariate backward regression analysis was carried out.

### Ethical considerations

Ethical approval was gained from the University of Nottingham Medical School Ethical Committee, the Jordanian Ministry of Health, the Jordan Royal Medical Services and the King Hussein Cancer Centre and permission to proceed with the study was secured. Participant informed consent, voluntary participation and confidentiality were guaranteed.

### Participants

Parents of a child with cancer were selected from four main public hospitals which are known to treat childhood cancer from the three main Jordanian cities (Amman, Irbid and Zarqa), plus the major military hospital in Jordan and The King Hussein Cancer Centre. Those cities cover more than 75 % of the Jordanian population [29] and these hospitals cover the wider geographical areas providing childhood cancer care across the entire country. Statistics have shown that these hospitals treat more than 85 % of the childhood cancer patients in the country [2]. Parents of school-based children from nine different schools located in the three major cities were approached so their responses could be considered as a base line of perceived stress levels for Jordanian parents.

### Measures

#### Perceived Stress Scale (PSS10)

The Perceived Stress Scale was selected as it reflects our theoretical position that stress occurs when perception of the demands exceeds the resources [35]. The questions used in the PSS10 questionnaire are all about demands and resources and the questions measure the participant’s feelings as to whether they have enough resources in comparison with the demands [36]. The questions are general in their nature and are judged over a broad spectrum in order to relate to participants with varying social circumstances (e.g. parents of a child with cancer and parents in the general population) [36]. Moreover, the questionnaire was translated into Arabic and used in Jordan before this study [37] and also more recently with good reliability and validity [38]. The possible range of scores is 0–40 with higher scores indicating greater perceptions of stress.

#### Family characteristics checklist

A family characteristics checklist was distributed to all parents and included: age of parent, gender of the parent, participant’s education level, participant’s work status, perception of family finances, number and age of children in the family, and health status of their children. An additional cancer-related checklist included additional information such as: distance from the hospital, age of the ill child, sex of the ill child, date of diagnosis, type of cancer, type of treatment.

#### Data analysis

Analyses were conducted using SPSS 17.0. Preliminary analyses included descriptives and then univariate inferential statistics were carried out using PSS10 scores as the outcome. To explore differences between the stress levels of parents with and without a child with cancer, adjusting for family characteristics, multivariate backward stepwise regression was carried. Dummy variables were used to enable inclusion of categorical predictors as appropriate. Separate multivariate backward stepwise regression analyses with further adjustment for cancer-related factors, were also carried out to explore differences in stress levels within the mother and father dyads caring for a child with cancer.

## Results

There were 405 couples caring for a child with cancer and who were invited to take part; 300 couples responded and were recruited to the study. In the school-based population it was not possible to ascertain how many families were approached via the schools, but 560 mothers and 550 fathers of children in the Jordanian schooling system responded, enabling 550 couples to be considered. 22 school-based couples were excluded as they reported that they had a child with a serious illness. The study sample reported here consisted of 300 couples caring for a child with cancer and 528 caring for children with no serious illness. Family characteristics are reported in Table 1 and were adjusted for in all initial multivariate models.

**Table 1:** Family characteristics

**(mean and SD)**	**Families - a child with cancer**		**Families - no children with serious illness**	
	**Mothers**	**Fathers**	**Mothers**	**Fathers**
Age	34.00 (7.31)	39.99 (8.09)	35.62 (5.83)	42.10 (6.66)
PSS10 scores	23.98 (8.21)	20.25 (8.68)	17.94 (6.41)	15.13 (7.12)
No. of children	2.84 (1.36)		3.56 (1.44)	
% of respondents				
Employed (unemployed)	11.0	90.0	26.3	93.9
Education				
Uneducated	1.0	0.3	0.4	0.0
Elementary school	7.0	9.7	8.0	9.7
High school	37.0	36.3	39.4	26.9
College	53.3	45.7	48.7	53.4
University	1.7	8.0	3.6	10.0
Comfortable	23.0	20.3	33.5	32.2
Varies	47.7	42.7	58.0	59.5
Tight	29.3	37.0	8.5	8.3

T-tests revealed that PSS10 scores were significantly different between fathers (p < 0.001) and between mothers (p < 0.001) from the two samples, with those in families with a child with cancer having significantly higher stress scores than those with no seriously ill children. PSS10 scores were also significantly different between mothers and fathers within couples (p < 0.001, paired t-test), with mothers reporting significantly higher stress levels than their husbands.

Multivariate analysis of the parental stress levels using backward stepwise regression analysis and adjusting for spousal stress and family characteristics was carried out on the whole sample (see Table 2). The final model for maternal stress (adjusted R^2^ =0.54, p < 0.001) showed a significant effect for having a child with cancer (p < 0.001) whilst adjusting for three additional predictors that remained significant, maternal age (p < 0.001), spousal stress (p < 0.001) and mothers’ perception of the family finances (p = 0.001 comfortable vs. tight, and p = 0.042 comfortable vs. varies). The final model for paternal stress (adjusted R^2^ =0.55, p < 0.001) showed no significant effect for having a child with cancer (p = 0.476) whilst adjusting for maternal employment status (p = 0.043), spousal stress (p < 0.001) and fathers’ perception of the family finances (p < 0.001 comfortable vs. tight, and p < 0.001 comfortable vs. varies) as significant predictors of maternal stress (adjusted R^2^ =0.52, p < 0.001). Exploration of this analysis identified that the cancer variable was non-significant only when spousal stress was in the model, but it became highly significant (p < 0.001) once spousal stress was removed (adjusted R^2^ =22, p < 0.001).

**Table 2:** All families - Final models for parental stress adjusting for spousal stress and family characteristics

	**Mothers**			**Fathers**		
	**(adjusted R**^**2**^ **= 0.54, p < 0.001)**			**(adjusted R**^**2**^ **= 0.52, p < 0.001)**		
	**β**	**t**	**p**	**β**	**t**	**p**
Constant	18.317	13.561	<0.001	3.969	2.699	0.007
Child with cancer yes/no	−2.575	−6.306	<0.001	−0.327	−0.713	0.048
Spousal stress	0.573	22.845	<0.001	0.660	23.064	<0.001
Father’s age			ns so removed			ns so removed
Mother’s age	−0.128	−4.446	<0.001			ns so removed
Father’s employment			ns so removed			ns so removed
Mother’s employment			ns so removed	−0.995	−2.024	0.043
Number of children			ns so removed			ns so removed
Own perception of family finances						
Comfortable vs varies	0.868	2.033	0.042	2.214	4.774	<0.001
Comfortable vs tight	1.980	3.232	0.001	4.660	7.236	<0.001

Within the couples who had a child with cancer, parental stress was analysed to identify whether different factors and covariates were associated with stress scores. In models which included spouse’s stress scores (see Table 3), family characteristics and cancer-related predictors 64 % of the variance was explained for mothers (adjusted R^2^ = 0.64, p < 0.001) and fathers (adjusted R^2^ = 0.64, p < 0.001), see Table 2. Models excluding spousal stress scores (see Table 4) explained just 26 % of the variance for fathers and 22 % for mothers.

**Table 3:** Cancer families only - Parental stress adjusting for spousal stress, family characteristics and cancer-related predictors

	**Mothers**			**Fathers**		
	**(adjusted R**^**2**^ **= 0.64, p < 0.001)**			**(adjusted R**^**2**^ **= 0.64, p < 0.001)**		
	**β**	**t**	**p**	**β**	**t**	**p**
Constant	15.326	10.507	<0.001	2.377	1.043	0.298
Spousal stress	0.700	19.617	<0.001	0.781	19.516	<0.001
Father’s age			ns so removed			ns so removed
Mother’s age			ns so removed	−0.218	−4.348	<0.001
Father’s employment	−2.305	−2.413	0.016	3.781	3.692	<0.001
Mother’s employment			ns so removed			ns so removed
Distance from home to hospital	−0.10	−2.498	0.013	0.010	2.421	0.016
Time since diagnosis	−0.031	−2.499	0.013			ns so removed
Age of ill child			ns so removed			ns so removed
Sex of ill child			ns so removed			ns so removed
Number of children	−0.640	−2.987	0.003	0.656	2.649	0.009
Own perception of family finances						
Comfortable vs varies			ns so removed			ns so removed
Comfortable vs tight			ns so removed			ns so removed
Type of cancer						
Lymphoma vs leukaemia			ns so removed			ns so removed
Lymphoma vs CNS tumour			ns so removed			ns so removed
Lymphoma vs other ca			ns so removed			ns so removed

**Table 4:** Cancer families - Parental stress adjusting for family characteristics and cancer-related predictors (excluding spousal stress)

	**Mothers**			**Fathers**		
	**(adjusted R**^**2**^ **= 0.23, p < 0.001)**			**(adjusted R**^**2**^ **= 0.26, p < 0.001)**		
	**β**	**t**	**p**	**β**	**t**	**p**
Constant	36.044	16.465	<0.001	27.264	9.625	<0.001
Father’s age			ns so removed			ns so removed
Mother’s age	−0.339	−5.537	<0.001	−0.409	−6.217	<0.001
Father’s employment			ns so removed	3.744	2.507	0.013
Mother’s employment			ns so removed			ns so removed
Distance from home to hospital			ns so removed			ns so removed
Time since diagnosis	−0.082	−4.551	<0.001	−0.074	−3.923	<0.001
Age of ill child			ns so removed			ns so removed
Sex of ill child			ns so removed	1.872	2.123	0.035
Number of children			ns so removed			ns so removed
Own perception of family finances						
Comfortable vs varies			ns but retained in model			ns but retained in model
Comfortable vs tight	2.498	2.105	0.036	3.479	2.77	0.006
Type of cancer						
Lymphoma vs leukaemia			ns so removed			ns so removed
Lymphoma vs CNS tumour			ns so removed			ns so removed
Lymphoma vs other ca			ns so removed			ns so removed

## Discussion

The school-based sample may be considered to be representative of mothers and fathers parenting well-children in the general Jordanian population and it provides descriptive data which can be used for PSS10 norm values of mothers and fathers in Jordan. Consequently, any research into the stress of parents who are parenting children with serious health conditions in Jordan could be informed by comparing their findings with the stress scores, in this study, of the mothers and fathers parenting children in school-based population.

The family characteristics included in the analysis covered most areas identified by Klassen et al. [28] (family social and economic characteristics, child characteristics, caregiverdemands, self perception, and coping). In families with or without a child with cancer, mothers reported higher stress levels than fathers. This is consistent with previous findings where female populations are known to report higher levels of stress than males [37,39,40]. Separate univariate analysis revealed that mothers and fathers in families with a child with cancer had significantly higher stress scores than mothers and fathers in families with well-children. This finding became more complex to interpret once adjustments were made for confounding variables. When carrying out analysis with paternal stress as the outcome, the cancer/well-child status was significant in univariate analysis and again in multivariate analysis when it excluded the maternal stress as a predictor, yet including the mothers’ stress score masked the effect of the cancer/well-child status. However, there is the important consideration that to exclude spousal stress can result in the loss of a predictor which is responsible for 30 % of the variance or more. Thus the decision whether or not to enter spousal stress score into a multivariate model needs to be recognised as having potential for considerable impact upon the variables likely to be significant. A recent American study of parents caring for a child with cancer has also identified that mother and father self-reports are positively correlated, but that mothers reported higher levels of stressors than fathers [41]. The models which include spouse stress levels and family characteristics explain 64 % of the parental stress of those caring for a child with cancer; however some aspects identified by Klassen et al. [28] were not able to be included in the models. Whilst child characteristics such as time since diagnosis and severity of illness were included, child’s own coping styles, psychological status, behaviour and quality life were not able to be incorporated into the research, nor was the area of individual parental coping styles. These are likely to contribute further to explaining parental outcomes and need to be considered in future research.

Our findings showed a significant effect of the spouse's stress score on parents and therefore healthcare providers need to consider the stress levels of both parents when planning interventions and support. The results indicated that parents of children with cancer require a support strategy based not only on the individual parental demands and resources but that health care providers should assess the family context of these parental demands and resources. This is supported by previous research which identifies that Arab parenting is collective “with fathers, mothers and other adults taking part in child rearing and socialization” [42].

## Conclusions

Both mothers and fathers with a child diagnosed with cancer reported higher stress levels than those from the normal Jordanian parent population. Mothers and fathers of children with cancer reported significantly different levels of stress to each other but models reveal significant contributions of the stress score of fathers upon mothers, and vice versa. Care should be taken when deciding whether to include wellbeing scores of a spouse/partner when analysing the wellbeing of a parent whose child has cancer. The findings provide evidence of the need for psychological support to be developed for families caring for a child with cancer in Jordan. Whilst Arab countries are not heterogeneous, Jordanian parenting, culture and religion will bear some similarities to other surrounding countries in the Middle East and therefore the results of this study might be beneficial not only for Jordanian parents but also for other Middle Eastern parents.

## Abbreviations

PSS10, Perceived Stress Scale 10 item.

## Competing interests

The authors declare that they have no competing interests.

## Authors’ contributions

RM conceived the research, carried out the literature review, led on the design of the study, was responsible for the data collection and initial analysis, and read and commented on all drafts of the manuscript and approved the final manuscript. JC contributed to the design of the study, participated in its co-ordination, carried out further analysis and drafted the manuscript. CH contributed to the design of the study, participated in its coordination, read and commented on all drafts of the manuscript and approved the final manuscript.
